# Waveguide integrated superconducting single-photon detectors with high internal quantum efficiency at telecom wavelengths

**DOI:** 10.1038/srep10941

**Published:** 2015-06-10

**Authors:** Oliver Kahl, Simone Ferrari, Vadim Kovalyuk, Gregory N. Goltsman, Alexander Korneev, Wolfram H. P. Pernice

**Affiliations:** 1 Institute of Nanotechnology, Karlsruhe Institute of Technology, Karlsruhe, 76132, Germany; 2 Department of Physics, Moscow State Pedagogical University, Moscow 119992, Russia; 3 Moscow Institute of Physics and Technology (State University), Moscow 141700, Russia; 4 National Research University Higher School of Economics, 20 Myasnitskaya Ulitsa, Moscow 101000, Russia

## Abstract

Superconducting nanowire single-photon detectors (SNSPDs) provide high efficiency for detecting individual photons while keeping dark counts and timing jitter minimal. Besides superior detection performance over a broad optical bandwidth, compatibility with an integrated optical platform is a crucial requirement for applications in emerging quantum photonic technologies. Here we present SNSPDs embedded in nanophotonic integrated circuits which achieve internal quantum efficiencies close to unity at 1550 nm wavelength. This allows for the SNSPDs to be operated at bias currents far below the critical current where unwanted dark count events reach milli-Hz levels while on-chip detection efficiencies above 70% are maintained. The measured dark count rates correspond to noise-equivalent powers in the 10^−19^ W/Hz^−1/2^ range and the timing jitter is as low as 35 ps. Our detectors are fully scalable and interface directly with waveguide-based optical platforms.

Highly efficient low noise single-photon detectors are key to the realization of numerous applications, both classical as well as quantum technological^1^,^2^. While classical applications such as optical time domain reflectometry and optical coherence tomography show a need for improved signal-to-noise ratio and detector timing characteristics^3^,^4^, quantum applications in quantum metrology and optical quantum computing crucially depend on high efficiency, low-noise performance and minimal timing jitter^5^,^6^,^7^,^8^,^9^,^10^,^11^. The implementation of numerous linear optics quantum information processing schemes, most prominently quantum key distribution, is currently limited by the availability of suitable quantum detectors in large numbers^1^,^6^,^7^,^8^,^12^. In the visible wavelength regime, silicon avalanche photodiodes (APDs) are available with good efficiency about 70%^13^. These devices provide, however, a limited optical operation bandwidth and are hard to integrate with chip-scale devices.

In order to be compatible with the existing telecommunication infrastructure it is desirable to operate in the near-infrared wavelength regime around 1550 nm. In this regime predominantly APDs based on the InGaAs material systems are employed^14^. Such detectors are, however, plagued by high dark count rates and detection efficiency below 30%. Furthermore, gated mode operation is prerequisite, thus limiting available detection rates and secure transmission distances^15^,^16^,^17^. As an alternative, superconducting nanowire single-photon detectors (SNSPD) have emerged as a promising detector architecture when ultra-low noise is required^18^. SNSPDs are operated at cryogenic temperatures below the critical temperature where the material is in its superconducting phase. Ohmic resistances are effectively zero in this state allowing currents below a specific critical current to flow unimpededly. The absorption of photons incident on the superconducting nanowire locally destroys the superconductivity which leads to a stark change in detector resistance which, in turn, can be registered electronically thus providing a clear signature of a single photon detection event. For applications in the telecommunication range SNSPDs have provided favorable performance characteristics, including timing jitter below 20 ps and dark count rates of only a few Hertz^19^,^20^,^21^. Traditional SNSPDs are coupled to optical fibers under normal incidence^22^,^23^,^24^. Because the nanowires are prepared from ultra-thin films with thicknesses below 5 nm, the available absorption length is limited by the film thickness, which allows a large portion of the incident photon flux to permeate the detector region without being absorbed. Therefore detection efficiencies have been on the order of 30% and are thus comparable to InGaAs APDs. Cavities can be used to recycle photons in order to boost the absorption as well as the detection efficiency^25^. However, this approach concomitantly reduces the optical bandwidth. Absorption under normal incidence is furthermore not compatible with an integrated optical platform where out-of-plane optical access is typically avoided. By using multi-layer thin-film architectures the absorption efficiency can be increased to 93%, as recently demonstrated at detectors made from tungsten silicon (WSi)^26^. This detector design nevertheless requires sophisticated fabrication approaches and is not easily scalable when multi-detector geometries are required in a single photonic system.

To overcome existing limitations in detector design, waveguide integrated nanowire^27^ superconducting detectors have been suggested as an alternative route^28^,^29^,^30^,^31^,^32^. In this design, photons are no longer absorbed under normal incidence, but rather by evanescent coupling to the superconducting nanowire which is situated directly atop an optical waveguide. The available coupling length over which photons are absorbed can in principle be increased arbitrarily to achieve near-unity absorption efficiency^33^. More importantly, however, such a design is directly compatible with integrated optical circuits and thus ideally suited to complement integrated quantum optical platforms^34^,^35^,^36^,^37^,^38^. Therefore, SNSPDs provide a crucial ingredient for integrated quantum optical systems. Waveguide integrated single photon detectors have demonstrated detection efficiency above 90%, while providing extremely low dark count rates in the milli-Hertz range^32^, paired with low timing jitter of 18 ps^39^. In the visible wavelength regime, detectors made from NbTiN have shown characteristic plateau behavior and thus high internal quantum efficiency^31^. For the important telecommunication regime, however, such devices are still sought after. High internal quantum efficiency is required in order to be able to operate the single photon detector far from the critical current which in turn provides low noise-equivalent power (NEP). Even though this has been achieved in the visible wavelength regime, where silicon APDs are available as alternative detectors^40^, the equivalent implementation in the near-infrared regime is missing.

Here we demonstrate highly efficient waveguide integrated SNSPDs with plateau behavior in the telecommunications wavelength range. Our detectors are made from niobium nitride (NbN) thin films deposited on top of silicon nitride (Si_3_N_4_) waveguides, thus also providing a low-loss waveguiding system with broad optical transparency^41^,^42^,^43^,^44^. We demonstrate detection efficiencies above 80% with NEPs at the 10^−19^  W/Hz^1/2^ level. Our devices are fully scalable and integrated into nanophotonic circuits, thus allowing for convenient combination with on-chip quantum optical circuits.

## Results

### Device fabrication and single-photon detector layout

**Figure 1:**
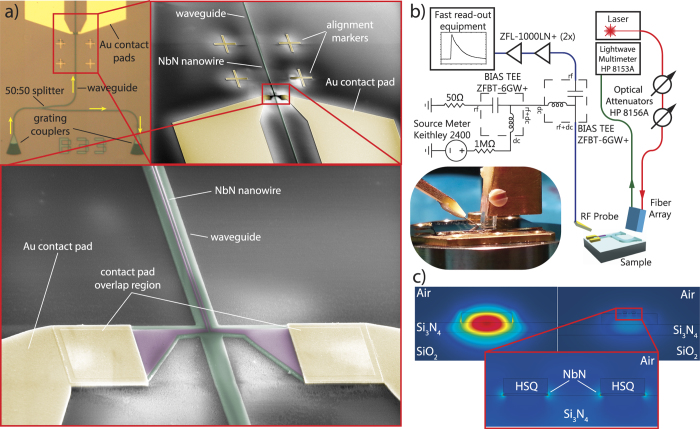
a) Optical microscope image of a nanophotonic circuit with integrated SNSPD device. Inset: SEM micrograph of the detector area with metal alignment mark and zoom into the waveguide region with NbN nanowire on top. **b**) Schematic of the experimental setup with combined optical and electrical access to the low-temperature probe chamber. **c**) Optical mode profile of the waveguide by itself (left) and the waveguide featuring NbN stripes patterned directly atop the waveguide (right) obtained through FEM simulations using COMSOL Multiphysics.

For the realization of nanophotonic circuits with broadband optical transparency we employ silicon nitride (Si_3_N_4_) as waveguiding material. Si_3_N_4_ provides low absorption loss in the telecommunications wavelength band, features low free-carrier absorption compared to silicon due to a large electronic bandgap and allows for tight optical confinement because of its relatively large refractive index^41^,^44^. Our SNSPDs are fabricated from 450 nm thick stoichiometric Si_3_N_4_ on insulator films with a 2.6 μm buried oxide layer. Prior to circuit fabrication on top of the nitride layer a 4 nm thick niobium nitride (NbN) film is deposited by magnetron sputtering in an Ar-N2 atmosphere^45^. The superconduction transition temperatures and square resistances of our NbN films aremeasured and found to be T_c_ = 8.6 ± 0.4 K and R_sq_ = 620 O hm/sq, respectively. In several nano-lithographical steps the waveguide and detector patterns are transferred into the respective chip layers and are electrically connected to metal contact pads (see methods section). The waveguides have been designed to support transverse-electrical (TE) mode-propagation at 1.55 μm wavelength. By placing the NbN nanowire directly into the optical near-field of the guided mode, light propagating along the waveguide experiences strong absorption along the nanowire. An optical microscope image and scanning electron beam micrographs of the completed design are shown in Fig. 1a). Electrical contact between the NbN layer and the gold contact pads is established by a large overlap region at the end of the nanowire. The nanowires are positioned with high accuracyusing metallic alignment markers which are realized in the first lithography step. Our detectors are fabricated as a single meander loop positioned in the center of the waveguide where the optical near-field intensity is maximal. The waveguides leading to the detectors are connected to grating couplers for optical input as shown in Fig. 1a). This way contact free optical characterization is possible for multiple detector circuits on the same chip^46^.

The devices are characterized inside a helium-4 flow cryostat with a base temperature of 1.7 K. The detector chip is placed on a multi-axis piezostage for accurate alignment with respect to an array of optical fibers and the contact fingers of an electrical RF-probe. Electrical read-out is enabled by bringing the RF-probe in physical contact with the gold contact pads on the chip. For optical access light is coupled into the waveguide circuit using the array of optical fibers which we position right on top of the grating couplers. Various electrical components are used to separate RF and DC contributions and amplify the detector signal (see Fig. 1b). Prior to the detector assessment, the circuits are characterized optically using transmission measurements. These allow us to precisely calibrate the photon flux travelling towards the detector region. The photons reaching the detector are then absorbed by the nanowire with high probability. Fig. 1c) shows the transverse-electric mode profile of the 1.5 μm wide 225 nm high rib waveguide with and without the NbN nanowire on top, obtained from FEM simulations using COMSOL Multiphysics. As can be seen, the larger portion of the electric field is concentrated around the NbN stripes when present, which illustrates the strong coupling of incident photons to the detector in our travelling waveguide SNSPD geometry. The large change in the imaginary part of the refractive index from the bare waveguide to the area including the NbN stripes leads to the desired strong absorption along the wire. Nanowires of 5 different widths (60, 80, 100, 120 and 140 nm), were fabricated, each with a gap of 120 nm between the stripes, yielding absorption rates of 0.06, 0.12, 0.19, 0.27, and 0.34 dB/μm, respectively. The propagation length of the wires, i.e. half the wire length for two-striped, single-loop wires, is varied from 40-90 μm in 10 μm increments, thus allowing us to investigate the detection efficiency in dependence of the wire length. Because the absorption efficiency increases exponentially with increasing wire length^33^, the overall detection efficiency improves accordingly. In the following we restrict our analysis to the longest wires where the absorption efficiency reaches its maximum value.

### Measurement of the on-chip detection efficiency

**Figure 2:**
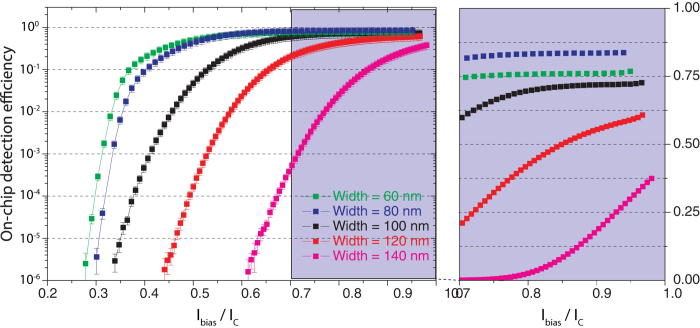
Measured on-chip detection efficiency as a function of normalized bias current for NbN nanowires of varying widths in logarithmic scale. The inset shows a zoom-in linear plot of the detection efficiencies close to *I*_c_ where the narrower nanowires (60, 80 and 100 nm) exhibit a plateau.

In order to investigate the performance of our SNSPDs the on-chip detection efficiencies (OCDE) are measured for various bias currents starting at the lowest current which still provides a measurable count signal. We use a low-noise variable current source and a bias-T to apply reproducible bias conditions as explained further in the methods section. The photon flux toward the detector is calibrated by adjusting the transmission through the nanophotonic circuit shown in Fig. 1a): light coupled into the waveguide at the grating coupler is split at a 50:50 Y-splitter to a second output grating coupler and the detector region. By recording the optical power at the reference port the optical intensity in the detector waveguide can be continuously monitored. We use optical attenuators to set the photon flux in the detector waveguide to an average value of 100 k counts per second. The detector count signal is recorded with counting electronics in dependence of the bias current. The current is slowly increased until the critical current I_C_ is reached and superconductivity is no longer sustainable. The resultant OCDE curves exhibit the usual sigmoidal shape (see fig. 2) with a maximum detection efficiency of ~90%. Saturation of the OCDE towards high bias currents becomes visible for narrower nanowires (60, 80 and 100 nm width) with the onset shifting to lower bias currents for narrower wires. The absolute OCDE increases for narrower wires. The 60 nm wide wire is an exception because here the absorption rate is lower as explained in the following.

Because the OCDE is the product of the detector’s internal quantum efficiency (QE) and its absorption efficiency (AE), OCDE = AE × QE, the maximum OCDE values measured are limited by either one or both of these contributions. In our travelling wave geometry, the AE of the SNSPD can be maximized by increasing the nanowire-covered area of the waveguide, i.e. by adjusting the width and the length of the detector^33^,^39^. The QE on the other hand inherently depends on the kinetics of the breakdown of the superconductivity upon the absorption of a photon^18^,^47^. The lower the energy conveyed by the photon, the smaller the size of the normal-conducting region resulting from photon absorption. Therefore, in order to detect near-infrared photons, nanowires of sufficiently narrow width are prerequisite for proper functionality. The interplay of these two contributions can be seen in Fig. 2: the OCDE near saturation increases from the wider wires to the narrower ones because of more favorable breakdown conditions. At the 60 nm wide wire, however, the lower AE due to the reduced nanowire-covered area becomes more dominant leading to a slightly reduced overall OCDE for this particular SNSPD.

More importantly, however, the narrower nanowires’ OCDEs saturate at bias currents well below I_C_, which is indicative of the QE approaching its maximum value: for a non-superconducting, resistive barrier to form, the bias-current must be sufficiently large to exceed the critical current density in the areas adjacent to the hotspot region. This is achieved either by reducing the nanowire width or increasing the bias current. From a certain point on, however, increasing the bias current will yield no further rise in OCDE. This point of high internal QE is reached at ~90% of I_C_ for the 100 nm wide nanowire, at ~80% of I_C_ for the 80 nm wide one, and at ~70% of I_C_ for the 60 nm wide one. This behavior in QE allows us to operate our detectors in a region where the internal QE is high while remaining sufficiently far from I_C_ to avoid the accumulation of false counts, which is attractive compared to alternative single-photon detectors at 1550 nm wavelength. The absolute OCDE values can then be further improved by extending the nanowire length to increase the AE^33^. Alternatively the waveguide cross-section could be reduced to achieve stronger coupling of propagating light to the nanowire.

### On-chip detection efficiency vs. noise-equivalent power

**Figure 3:**
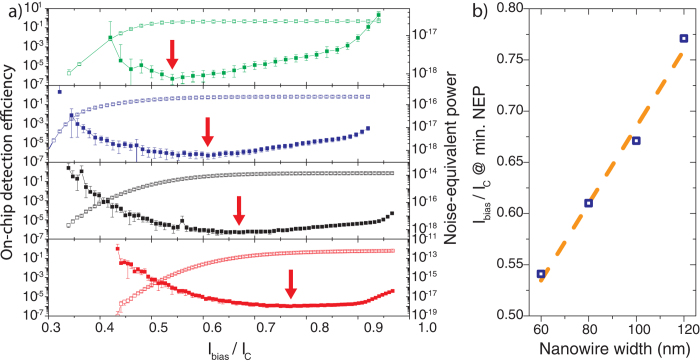
**a**) On-chip detection efficiency (open squares) and noise-equivalent power (closed squares) for 60 nm wide (green), 80 nm wide (blue), 100 nm wide (black) and 120 nm wide (red) nanowires as a function of normalized bias current. The red arrows indicate the minimal noise-equivalent power. **b**) Normalized bias current at minimal noise-equivalent power plotted versus nanowire width shows clear trend of minimal NEP shifting to lower bias currents for narrower wires.

Besides high OCDE, a single-photon detector should count as little unwanted events as possible. The quantity we use to characterize this performance aspect is the noise-equivalent power (NEP), which is defined as 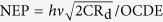, where *hν* is the photon energy and CR_d_ is the dark count rate. Fig. 3a) shows the OCDE and pertinent NEP curves for SNSPDs of 60, 80, 100, and 120 nm width. The NEP curves exhibit a convex functional dependence on the normalized bias current with their minima shifting to lower bias currents for narrower wires as indicated by the red arrows in Fig. 3a) and illustrated in Fig. 3b). This characteristic together with the early onset of the OCDE plateau for the narrower nanowires can be exploited to lower the operating bias current in order to reduce the NEP at no or little loss in OCDE. In the case of the 80 nm wide SNSPD, we achieve >70% OCDE at the 10^−19^ W/Hz^−1/2^ level with a bias current of 61% of I_C_^48^.

**Figure 4:**
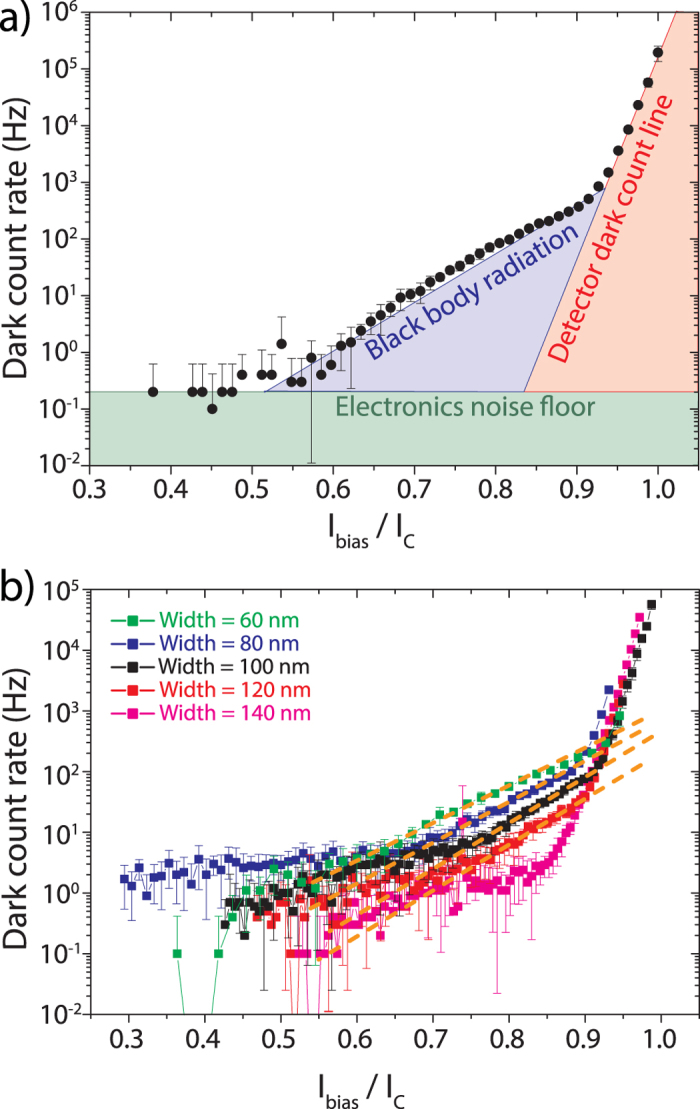
**a**) Dark count rate as a function of normalized bias current showing the major contributions to the experimentally measured dark count curves, i.e. false counts due to residual black body radiation and electronic noise. **b**) Dark count rate as a function of normalized bias current for nanowires of various nanowire widths. The orange dashed lines illustrate the growing contribution of black body radiation for narrower wires.

The accuracy with which we are able to determine the NEP is limited by our experimental conditions, such that the real NEP is, in fact, lower than the one measured and portrayed here. We conclude this from measuring the dark count rate for SNSPDs of different widths and analyzing the three salient contributions: the intrinsic detector dark count line, the electronics noise floor, and contributions from stray light entering the detector. The dark count rate curve displayed in Fig. 4a), measured with an 80 nm wide SNSPD with a length of 80 μm, serves as an example where the individual contributions are easily discernable. In the low biasing current regime, a current-independent noise floor (green) originates from imperfect shielding and the weak galvanic isolation of our equipment (green), which can be lowered by the installation of appropriate equipment. In the high current regime, the steep, strongly current-dependent contribution (orange) is due to fluctuations in the superconducting phase which can be thermally activated when close to the superconducting transition or which result from quantum decoherence effects, so called quantum phase slips^49^,^51^. It follows an exponential trend given by *CR*_*d*_ ∝ exp (*I*_*bias*_*h*/4*ek*_*B*_*T*)^49^,^50^, where *h* is Planck’s constant, *e* the electron’s elementary charge, and *k*_*B*_ Boltzmann’s constant. Lastly, a moderately current-dependent addition (purple) arises from stray light entering the detector which we expect to be mostly composed of black-body radiation in our otherwise dark laboratory. We verify this by comparing the dark count rate measured when the fiber array is mounted directly above the grating couplers to that when the fiber array is distant from the couplers and the input fibers are disconnected and shielded by metal caps at the input. As no measurable difference was found, we conclude that no residual visible room light enters our system. Additionally, we analyze the extent of the contribution of black body radiation to differently wide SNSPDs: as narrower wires are more susceptible to photons of lower energy, these detectors should show a larger stray light contribution. Fig. 4b) shows the measured dark count rate curves for SNSPDs of 60, 80, 100, 120, and 140 nm width. As indicated by the dashed orange lines, the amount of black-body radiation grows with decreasing wire width, thereby confirming our assumption. The widest nanowire detectors (magenta curve)only shows an exponential decay with decreasing bias current towards the electronics noise floor, indicating that for this wire width the black body photon energy is insufficient to trigger a detection event. While these dark counts limit our NEP values in the higher bias current region they could be reduced by installing appropriate shielding equipment inside the cryostat and using better isolated electronics.

### Timing jitter and decay time

**Figure 5:**
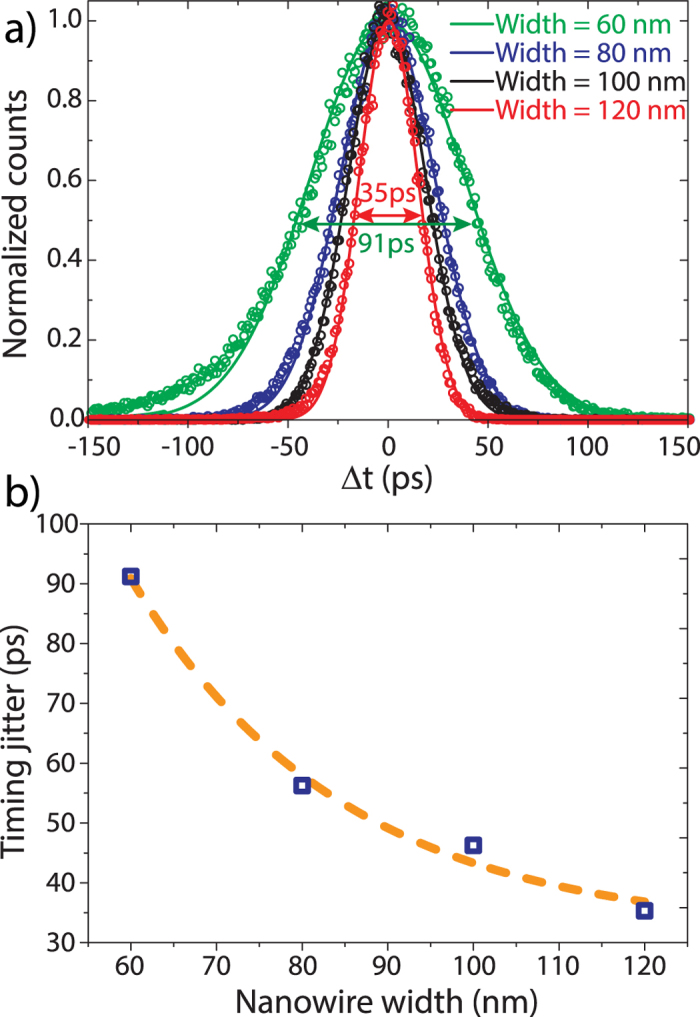
**a**) Histogram data obtained during the jitter measurement of various nanowire widths (empty circles) and corresponding Gaussian fit (solid line). The double-arrows indicate the timing jitter, i.e. the full width at half maximum (FWHM) obtained from the fits. **b**) Timing jitter as a function of nanowire width showing decreasing timing jitter with growing nanowire width.

Not only high efficiency and low-noise performance are required of SNSPDs, but also superior timing characteristics e.g. in order to unambiguously distinguish optical paths in time-bin measurements in large-scale quantum optical circuitry^52^. Measuring with high timing accuracy and high detection efficiency require the detector to be operated on the plateau area. In Fig. 5a) we show the experimentally obtained histograms of SNSPDs of various widths measured at a bias current of 95% of the critical current. For our measurements we use a pulsed laser source with internal timing jitter below 1 ps as a timing reference. The light from the pulsed laser is divided by a 50:50 beam splitter, one path of which leads to a fast detector outside the cryostat to provide the reference trigger. The second path guides light towards the on-chip detector. The arrival times of the detector clicks are recorded with a digital sampling oscilloscope in histogram mode. The timing jitter is extracted from the histograms by a Gaussian fit. All measured devices show timing jitters below 100 ps with the widest SNSPD reaching 35 ps. A trend of the timing jitter growing with decreasing wire width is clearly visible (Fig. 5b).

**Figure 6:**
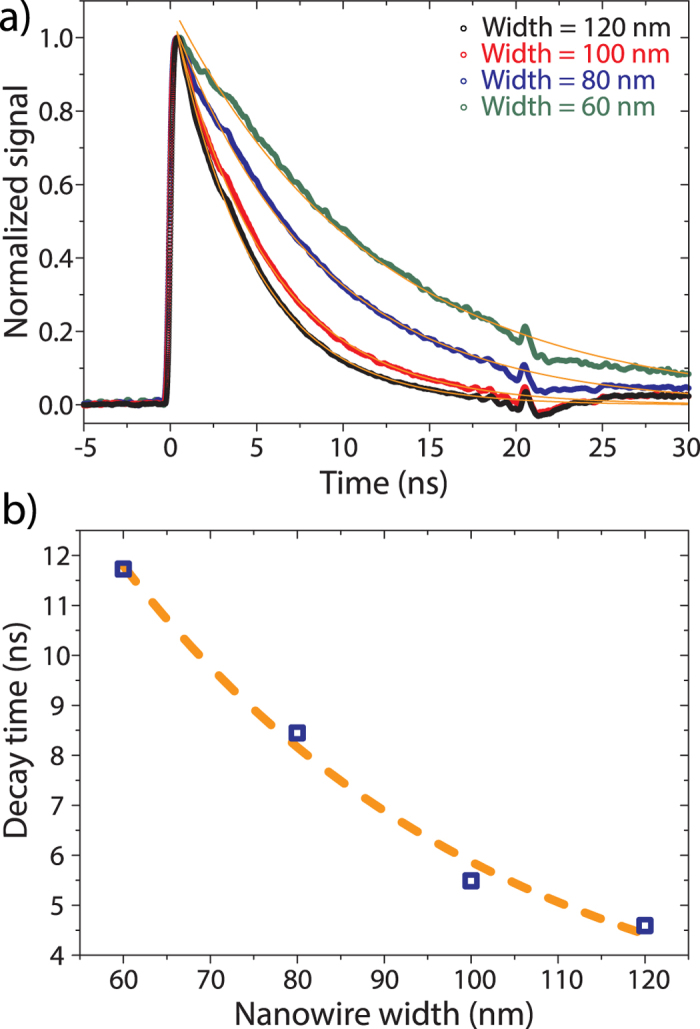
**a**) Measured pulse shape for various nanowire widths. **b**) Detector decay time as a function of nanowire width. The dashed line shows a hyperbolic fit, illustrating the inverse proportionality to wire width as expected for kinetic-inductance-limited SNSPDs.

The associated, averaged signal traces normalized to their respective maxima are depicted in Fig. 6a). Single-exponentials are fitted to the traces to extract the decay times which follow a clear trend from ~5 ns in the case of the 120 nm wide SNSPD to ~12 ns for the narrowest, 60 nm wide detector. A hyperbola fitted to the obtained decay time values indicates reciprocal dependence on the nanowire width (see Fig. 6b). This functional dependence is expected of SNSPDs whose speed is limited by their kinetic inductances 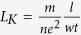
53, where *m* is the electron mass, *n* the charge carrier density, *e* the elementary charge of the electron, and *l*, *w*, and *t* the length, width and thickness of the superconducting nanowire, respectively. Therefore, the decay time of our detectors remains well within a 20 ns window. The measured decay times and timing jitters are slightly larger than those of comparable NbN-on-Si SNSPDs, but still smaller than those of most externally operated units like PMTs and SPADs^1^,^14^,^54^,^55^. We attribute the difference to the fact that in our case NbN is deposited onto amorphous substrates, rather than crystalline material as in the case of silicon. We expect that the quality of our SNSPDs can be further improved by refining the fabrication steps, resulting in higher critical current values which in turn will produce higher output pulses and hence reduce timing jitter.

## Discussion

The waveguide integrated single photon detectors demonstrated here show attractive performance characteristics in terms of on-chip detection efficiency, low dark count rate and low timing jitter. Particularly interesting is the fact that narrow nanowires with design widths below 100 nm show a clear plateau behavior towards high bias currents. This enables our detectors to be operated well below the critical current which, in turn, provides a favorable combination of low dark count rates and high detection efficiency. This synergy is expressed in low noise-equivalent power values obtained at a bias point as low as 61% of I_C_. While reducing the dark count rate by lowing the SNSPD’s bias current has little or no effect on the achievable detection efficiency, we found that at lower bias currents the timing jitter tends to increase. Therefore, a small trade-off between timing accuracy and low-noise performance exists. This trade-off has to be taken into account when selecting a detector geometry for a particular application.

Waveguide integrated detector devices therefore feature all three detector characteristics that are required for advanced applications in a single device^1^,^2^,^12^. In addition, the nanowire detectors provide a small footprint and a short overall length due to the single meander design. These features make the detectors presented here ideal candidates for applications in on-chip quantum optics and chip-scale metrology^5^,^10^. Paired with the high timing resolution provided by the low timing jitter, such waveguide detectors may find use in a number of applications ranging from correlation measurements to timing analysis and optical-time-domain reflectometry^3^,^4^. Because of the scalability of our fabrication approach, a multitude of such detector circuits can be realized in a single fabrication run, thus also holding promise for applications where multiple detectors are required in a single device.

In conclusion we have demonstrated silicon nitride nanophotonic circuits equipped with high performance NbN superconducting single photon detectors. Our devices provide high detection efficiency in a range of nanowire geometries, allowing for tailoring the detector layout to specific needs. Owing to its broadband transparency extending from the ultraviolet to mid-infrared wavelengths, the employed waveguide material Si_3_N_4_ provides a promising platform for non-classical optical applications, advanced sensing and single photon imaging.

## Methods

### Device fabrication

The nanophotonic circuits including detector devices are realized with three subsequent lithography steps. The first fabrication step includes electron beam lithography of the electrical contacts and alignment marker patterns for the nanowires and waveguides using polymethyl methacrylate (PMMA) as a positive resist. After development, a 5 nm thick Cr adhesion layer and 150 nm of Au are deposited onto the sample by electron beam physical vapor deposition (ePVD), which is directly followed by lift-off in acetone to finalize the contact pads and alignment markers. 5 nm of SiO_2_ are subsequently vapor deposited to provide adhesion for hydrogen silsesquioxane (HSQ) resist which is employed in a second electron beam lithography step to form the nanowire pattern. The wire consists of two long, parallel stripes connected with a 180 degree bend on the one end and larger patches for electrical connection on the other. After development, the SiO_2_ layer is removed by reactive ion etching (RIE) using an Ar plasma and the nanowire pattern is transferred into the NbN layer by RIE using a CF_4_ plasma. Finally, the waveguide pattern is written into ma-N 2403 resist by electron beam lithography and transferred into the underlying Si_3_N_4_ layer by a carefully timed RIE step using a CHF_3_ plasma to obtain 225 nm high rib-waveguide structures. Residual ma-N 2403 resist is removed by an additional O_2_ plasma step.

### Measurement setup

All measurements are performed inside a liquid helium cryostat at 1.7 K temperature. A sample holding contraption comprising four nanopositioners (x, y, z, rot), an array of optical fibers, and an RF-probe are utilized to access multiple devices on a single chip. The sample is mounted on top of the nanopositioners which enable us to position devices directly below the fiber array, such that optical signals can be routed onto and collected from the chip through optical grating couplers patterned into the Si_3_N_4_ layer. In a similar fashion, electrical contact is established by bringing the RF-probe in physical contact the sample’s contact pads. Light from a tunable laser source (New Focus TSL-6600/ PriTel FFL-40 M) is attenuated using two optical attenuators (HP 8156 A), capable of 120 dB total attenuation, to produce a specific photon flux in the waveguide. The input and output optical power is monitored by an optical power meter (HP 5153 A Lightwave Multimeter). The bias current to the SNSPD is supplied by a Keithley 2400 source meter operated in voltage mode and a 1 Mohm series resistor. We use two MiniCircuits Bias-Tee ZFBT-GW6+to filter and separate RF and DC contributions to and from the SNSPD, and two ZFL-1000LN+to amplify the detector signal. The signal is analyzed by an Agilent Infiniium 54855 A DSO oscilloscope and an Agilent 53132 A Universal Counter is used for photon number counting. Before every measurement, the nanowires’ critical current is measured, which ranges from 6 μA to 20 μA depending on the wire’s width.

### Optical and electrical characterization

The OCDE is determined by measuring the SNSPD count rates at a constant, high photon flux inside the waveguide (“light count rate”) as well as without injecting any light into the waveguide (“dark count rate”). The OCDE is then the ratio of the “true” count rate (light count rate less dark count rate) and the number of photons incident on the detector. We estimate an overall one-sigma error contribution of at most 20% in the photon flux toward the detector, which comprises variations in the grating coupler transmission and the 50:50 waveguide splitters. Variations in the photon counting equipment are accounted for by repeated measurement of the same data point. The detector timing jitter is determined by using a fiber coupled, pulsed laser source (PriTel FFL-40 M) producing pulses of ~1 ps duration at a repetition rate of 40 MHz. The light from the laser is split into two channels by a 50:50 fiber splitter. One channel leads to the SNSPD, which causes a constant count rate of 40 MHz, while the other channel is connected to an ultra-fast photo-receiver (New Focus 1611) which produces a similar electrical signal of 40 MHz. Both electrical signals are connected to an ultrafast digital sampling oscilloscope (Agilent Infiniium 54855 A DSO) operated in histogram mode. The trigger level is set to half the detector output voltage pulse magnitude and the arrival times of at least 1,000 pulses are collected. A Gaussian is then fitted to the resultant histogram, whose full width at half maximum (FWHM) represents the timing jitter. The intrinsic instrument jitters of the oscilloscope, laser, and photo-receiver are significantly lower (<2 ps) than the one measured at the SNSPD such that only a small error contribution is incurred.

## Additional Information

**How to cite this article**: Kahl, O. *et al*. Waveguide integrated superconducting single-photon detectors with high internal quantum efficiency at telecom wavelengths. *Sci. Rep*. **5**, 10941; doi: 10.1038/srep10941 (2015).
